# Symbiotic bacteria motivate the foraging decision and promote fecundity and survival of *Bactrocera dorsalis* (Diptera: Tephritidae)

**DOI:** 10.1186/s12866-019-1607-3

**Published:** 2019-10-22

**Authors:** Mazarin Akami, Xue-Ming Ren, Xuewei Qi, Abdelaziz Mansour, Bingli Gao, Shuai Cao, Chang-Ying Niu

**Affiliations:** 1 Department of Plant Protection, College of Plant Science & Technology, Huazhong Agricultural University, Wuhan, 430070 China; 2 Department of Biological Sciences, Faculty of Science, University of Ngaoundere, P.O Box 454, Ngaoundere, Cameroon; 3 Department of Economic Entomology and Pesticides, Faculty of Agriculture, Cairo University, Giza, 12613 Egypt

**Keywords:** *Bactrocera dorsalis*, Probiotics, Foraging behavior, Survival, Fecundity

## Abstract

**Background:**

The gut bacteria of tephritid fruit flies play prominent roles in nutrition, reproduction, maintenance and ecological adaptations of the host. Here, we adopted an approach based on direct observation of symbiotic or axenic flies feeding on dishes seeded with drops of full diet (containing all amino acids) or full diet supplemented with bacteria at similar concentrations to explore the effects of intestinal bacteria on foraging decision and fitness of *Bactrocera dorsalis*.

**Results:**

The results show that intestinal probiotics elicit beneficial foraging decision and enhance the female reproduction fitness and survival of *B. dorsalis* (symbiotic and axenic), yet preferences for probiotic diets were significantly higher in axenic flies to which they responded faster compared to full diet. Moreover, females fed diet supplemented with *Pantoea dispersa* and *Enterobacter cloacae* laid more eggs but had shorter lifespan while female fed *Enterococcus faecalis* and *Klebsiella oxytoca* enriched diets lived longer but had lower fecundity compared to the positive control. Conversely, flies fed sugar diet (negative control) were not able to produce eggs, but lived longer than those from the positive control.

**Conclusions:**

These results suggest that intestinal bacteria can drive the foraging decision in a way which promotes the reproduction and survival of *B. dorsalis*. Our data highlight the potentials of gut bacterial isolates to control the foraging behavior of the fly and empower the sterile insect technique (SIT) program through the mass rearing.

## Background

Insects are capable of shaping their foraging behavior and food consumption in a way that favors their growth and reproduction [1–3]. In this light, the substrate specific chemoreceptors are key factors in the responses of insects to environmental stimuli such as food and whose latency to respond depends on the nutritional status of the insect [4–6].

Many insects are associated with diverse extracellular microorganisms that can be found, among other sites on the exoskeleton, in the hemocoel, or in the gut lumen [7], and with intracellular microorganisms that populate specialized tissues or organs such as bacteriocytes [8]. Their relationships with their hosts are often linked to their status as intra or extra-cellular symbionts and range from parasitic to mutualistic [9–11]. The intra-cellular symbionts are often considered as obligate ones, for, they cannot live outside the host, so they are transmitted vertically (from mother to progeny) [12]. Both, intra and extra-cellular symbionts play a variety of functions on host biology, survival and foraging activity [13–18]. For example, the gut microbiome of the vinegar fly *Drosophila melanogaster* was shown to indirectly influence the foraging behavior of the host by modulating their immune system, lipid and carbohydrate accumulation [19–21] and olfactory sensitivity for the own benefits of bacteria [22]. Gut bacteria was shown to be implicated in the resistance and susceptibility of *Callosobruchus maculatus* to dichlorvos and essential oil [23]. Intestinal probiotic *Klebsiella oxytoca* (member of Enterobacteriaceae family) restored the ecological fitness of irradiated *B. dorsalis* males by promoting food intake and metabolic activities [24].

Furthermore, some insects possess the ability to cultivate and digest their own gut bacteria as additional protein source to fuel their metabolic functions [25, 26]. These evidences somehow show that gut bacteria and the host nutritional status are intimately associated in driving the fitness and foraging behavior of the insect [18].

Tephritidae fruit flies (Diptera: Tephritidae) harbour bacterial communities dominated by species of Enterobacteriacae [27]. These microbes have been shown in other fruit flies to be involved in host longevity [28, 29], nitrogen fixation [30], reproductive success [31, 32], protection from pathogens [33] and detoxification of xenobiotics [27, 34–36]. In order to survive and reproduce, these flies should acquire nutrients (carbohydrate and protein) from the environment through their foraging activity. The presence of gut microbiome in adult flies contributes to their nutrition by providing essential amino acids missing from their diets. For example, symbiotic olive flies *Bactrocera oleae* have been able to produce eggs when fed only non-essential amino acids, while aposymbiotic flies have been unable to do so [28, 29]. Moreover, bacteria supplemented diets were shown to increase the life expectancy and fecundity of the flies in comparison to normal diets. For instance, female olive flies fed sugar diet inoculated with *Pseudomonas putida* laid more eggs than those fed sugar diet only [37]. Similarly, *Enterobacter agglomerans* and *Klebsiella pneumoniae* improved the dietary outcomes of yeast-based foods that positively affected the longevity and female reproductive capacity of the Mediterranean fly *Ceratitis capitata* [38].

The oriental fruit fly *Bactrocera dorsalis* (Diptera: Tephritidae) is a serious pest which causes considerable loss of cultivated crops worldwide and attacks over 350 host species [39, 40]. The bacterial populations inhabiting the gut and reproductive organs of this pest were shown to play important roles in host physiology and behavior [39, 41–43].

In the present study, we assessed the effects of four bacterial isolates on foraging choice and fitness of *B. dorsalis*. We hypothesized that intestinal bacteria motivate the foraging decision and enhance the reproduction fitness and survival of the fly. The method consisted of offering to protein starved flies, symbiotic or axenic, a choice between full diets (containing all amino acids, sugar and minerals) or full diets supplemented with individual bacterial isolates (*Pantoea dispersa*, *Enterobacter cloacae, Enterococcus faecalis* and *Klebsiella oxytoca*). In the first experimental setting, we evaluated the effects of the presence or absence of bacteria on the responses of the flies (landing latency, food choice and ingestion) to the diets presented, while in the second experiment, we evaluated the fecundity and longevity of females fed full and probiotic diets, respectively. We predicted that, when deprived of their gut bacteria (axenic), the flies would consistently chose the most profitable diet to sustain their maintenance and reproduction fitness.

## Results

### Effects of bacterial isolates (probiotics) and symbiotic status on the foraging decision of *B. dorsalis*

Here, we evaluated the overall response of the flies (symbiotic and axenic) to the diets, irrespective of their quality (full diet only or full diet + bacterial isolates). Then we compared the landing events on probiotic diets of axenic flies to those of symbiotic ones together with the time elapsed to the first landing decision (latency) in both flies.

**Fig. 1:**
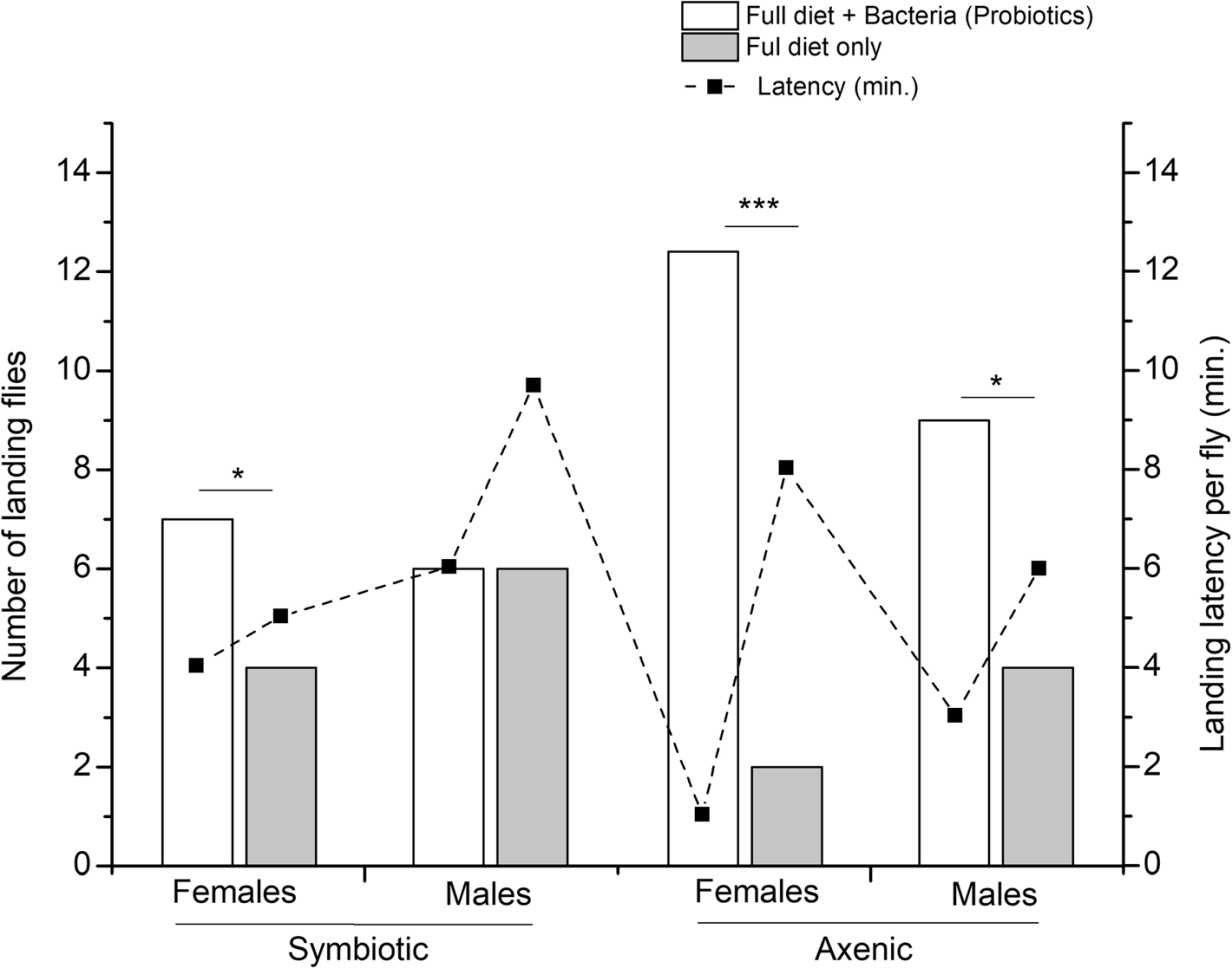
Response of symbiotic and axenic *Bactrocera dorsalis* to experimental diets (full amino acids diet or full diet + bacteria isolate). The left ordinate is the average number of flies which landed and the right ones correspond to their latency to respond. Each bar represents the marginal number of flies which landed on food patch (regardless of food quality) within an hour of observation. Means were separated with Student’s t-Test at *P* = 0.05

Most of the experimental flies tested responded positively by landing on the diets presented. Of the 15 axenic females and 15 males tested in each treatment, 12 and 8 flies, respectively, landed on patches containing probiotic diets compared to symbiotic ones (χ^2^ = 4.756, df = 3, 57, *P* < 0.0001 and χ^2^ = 33.33, df = 3, 57, *P* = 0.0029, respectively) (Fig. 1 ). Diets containing bacteria (probiotics) elicited more landings than those containing full diets only. With the exception of symbiotic males which showed no landing preference to either diets (ANOVA, F = 77.34, df = 1, 59, *P* = 0.707), all the experimental flies landed at significantly higher rates on probiotic diets as compared with full diets only (ANOVA, Axenic females: F = 210.80, df = 1, 59, *P* < 0.0001; Axenic males: F = 39.347, df = 1, 59, *P* = 0.001 and symbiotic females: F = 77.34, df = 1, 59, *P* < 0.001) (Fig. 1).

In general, axenic flies responded faster in the experimental chambers than the symbiotic flies, and landings on the full diets inoculated with bacteria occurred faster than landings on the full diets only (Chi-square test, χ^2^ = 7.93, R^2^ = 0.998, *P* = 0.001) (Fig. 1). Axenic females and males landed within 1 and 3 min post-presentation on probiotic diets, respectively. Conversely, latency to land on full diets was longer in axenic females than in the symbiotic ones (ANOVA, F = 11.834, df = 1, 59, *P* < 0.0001) (Fig. 1).

### Food consumption

**Fig. 2:**
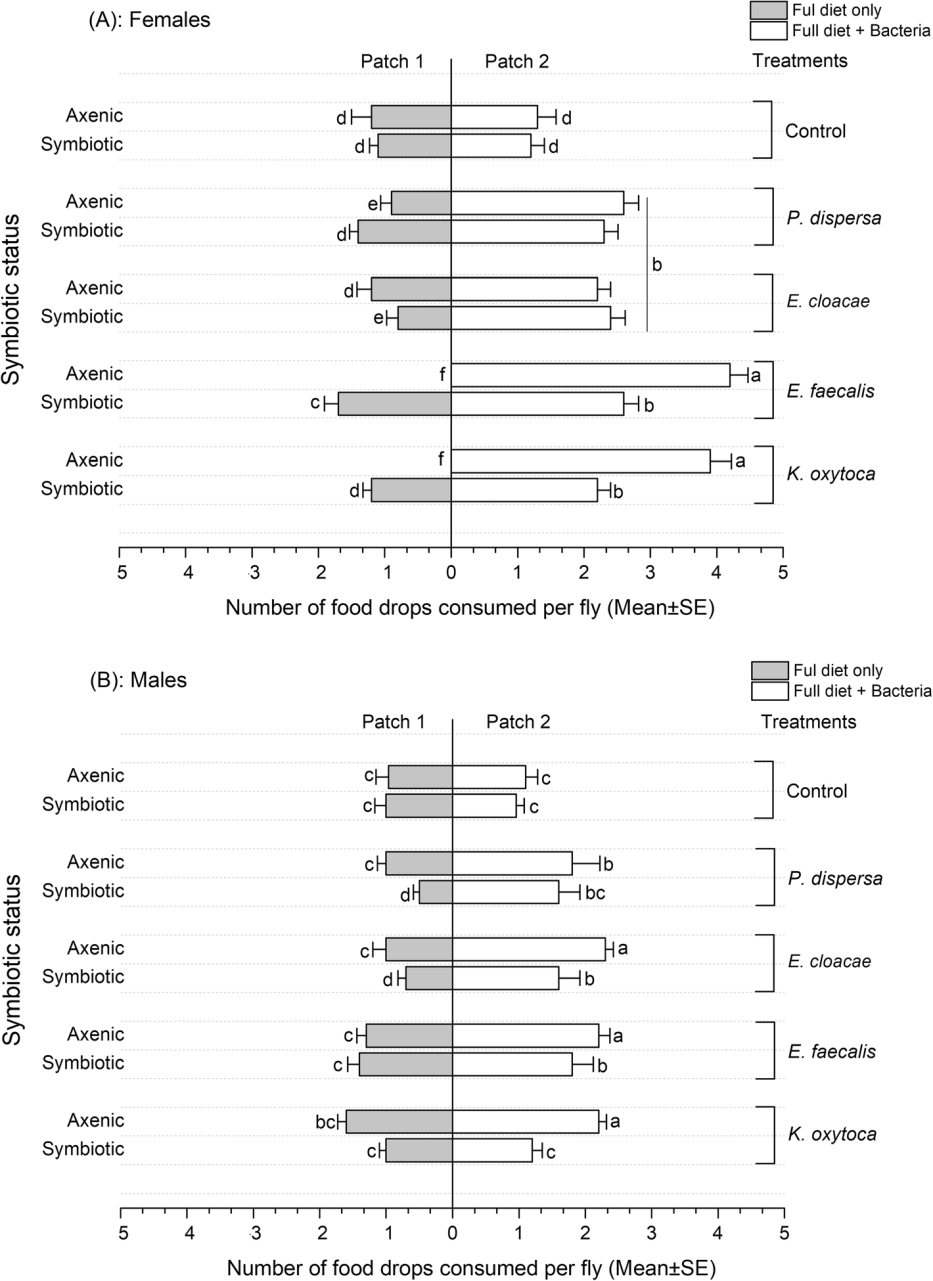
Number of nutritional drops consumed by females (**a**) and males (**b**) symbiotic and axenic flies (*Bactrocera dorsalis*) exposed to two diet patches (containing full diet and full diet + bacteria isolate, respectively). Each horizontal bar represents the Mean ± SE of drops consumed by symbiotic and axenic flies from each treatment group within an hour of observation

The diet composition, sex and symbiotic status of the flies affected significantly the number of drops consumed by the experimental flies (Regression Model, F = 15.834; df = 2, 58; R^2^ = 0.983; t = 6.048; *P* < 0.001) (Fig. 2).

In general, axenic flies (females and males) consumed more food drops than the symbiotic ones (ANOVA, F = 19.34, df = 3, 57, *P* < 0.0001, and F = 16.761, df = 3, 57, *P* = 0.001, for females and males, respectively), except in the control groups where the consumption of both diet patches was similar in females and males (ANOVA, F = 13.40, df = 3, 57, *P* = 0.554, and F = 24.03, df = 3, 57, *P* = 0.658, respectively) (Fig. 2 a & b). Overall, female flies consumed more of the food drops presented than the male ones in all treatments (ANOVA, F = 17.376, df = 1, 59, *P* < 0.0001).

Ingestion of probiotic diets was significantly higher in all tested flies, males and females (ANOVA, F = 13.81, df = 3, 57, *P* = 0.004 and F = 37.25, df = 3, 57, *P* < 0.0001, respectively) (Fig. 2 a & b). Nevertheless, the axenic flies (females and males) displayed a significantly higher preference toward full diet supplemented with bacteria isolates compared to symbiotic flies (F = 65.14, df = 4, 56, *P* < 0.0001 and F = 11.41, df = 4, 56, *P* < 0.0001, respectively) (Fig. 2 a & b). Axenic female flies consumed numerous drops of full diet inoculated with *E. faecalis* and *K. oxytoca*, compared to those supplemented with *P. dispersa* and *E. cloacae* (F = 21.815, df = 4, 56, *P* < 0.0001 and F = 12.693, df = 4, 56, *P* < 0.0001, respectively) (Fig. 2a) and compared to the control (F = 46.206, df = 4, 56, P < 0.0001 and F = 35.263, df = 4, 56, *P* < 0.0001, respectively) (Fig. 2a). Similarly, axenic male flies ingested more drops of *E. faecalis, K. oxytoca* and *E. cloacae* supplemented diets compared to *P. dispersa* enriched diet and the control, respectively (F = 10.724, df = 4, 56, *P* < 0.001 and F = 30.810, df = 4, 56, *P* < 0.001, respectively) (Fig. 2b).

### Bacterial effects on fitness parameters

#### Female fecundity

**Fig. 3:**
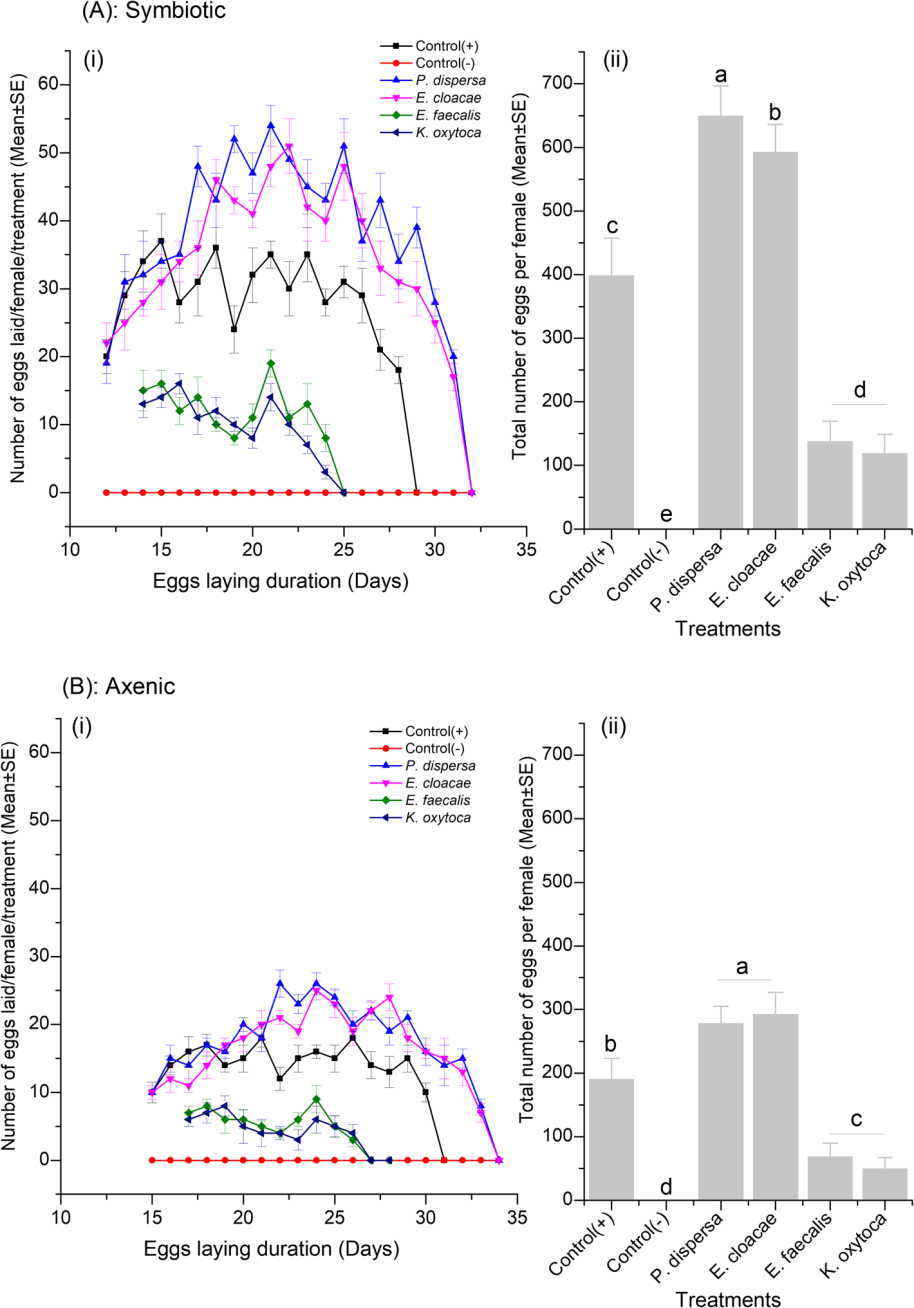
Average number of lifelong eggs laid by the female *B. dorsalis*. The control group was fed full diets only. (**a**): Symbiotic flies and (**b**): Axenic flies; (i): daily number of eggs laid by each female fly & (ii): average lifelong eggs lay per female. Mean bars with different letters between treatments are statistically different after Tukey’s post hoc tests at *P* ≤ 0.05

Bacterial isolates of *P. dispersa* and *E. cloacae*, significantly increased egg productions in symbiotic and axenic *B. dorsalis* females from the fifth feeding day as compared with the positive control (ANOVA, F = 111.351, df = 5, 55, *P* < 0.0001 and F = 177.404, df = 5, 55, *P* < 0.001, respectively) (Fig. 3 a & b). Conversely, symbiotic and axenic females fed *E. faecalis* and *K. oxytoca* enriched diets drastically reduced the lifelong number of eggs laid compared to positive control (F = 45.297, df = 5, 55, *P* < 0.0001 and F = 177.404, df = 5, 55, *P* < 0.0001, respectively) and remained lower throughout the experimental period (Fig. 3 a & b). When *B. dorsalis* females were fed only sugar diet (negative control), they were not able to produce eggs irrespective of their symbiotic status. The fecundity capacity of symbiotic females fed *E. faecalis* and *K. oxytoca* enriched diet was not different from each other (F = 45.297, df = 5, 55, *P* = 0.587) (Fig. 3a) but remained higher than that of the axenic females fed the same bacterial diets (F = 45.297, df = 5, 55, *P* < 0.001) (Fig. 3b).

#### Female life expectancy

**Fig. 4:**
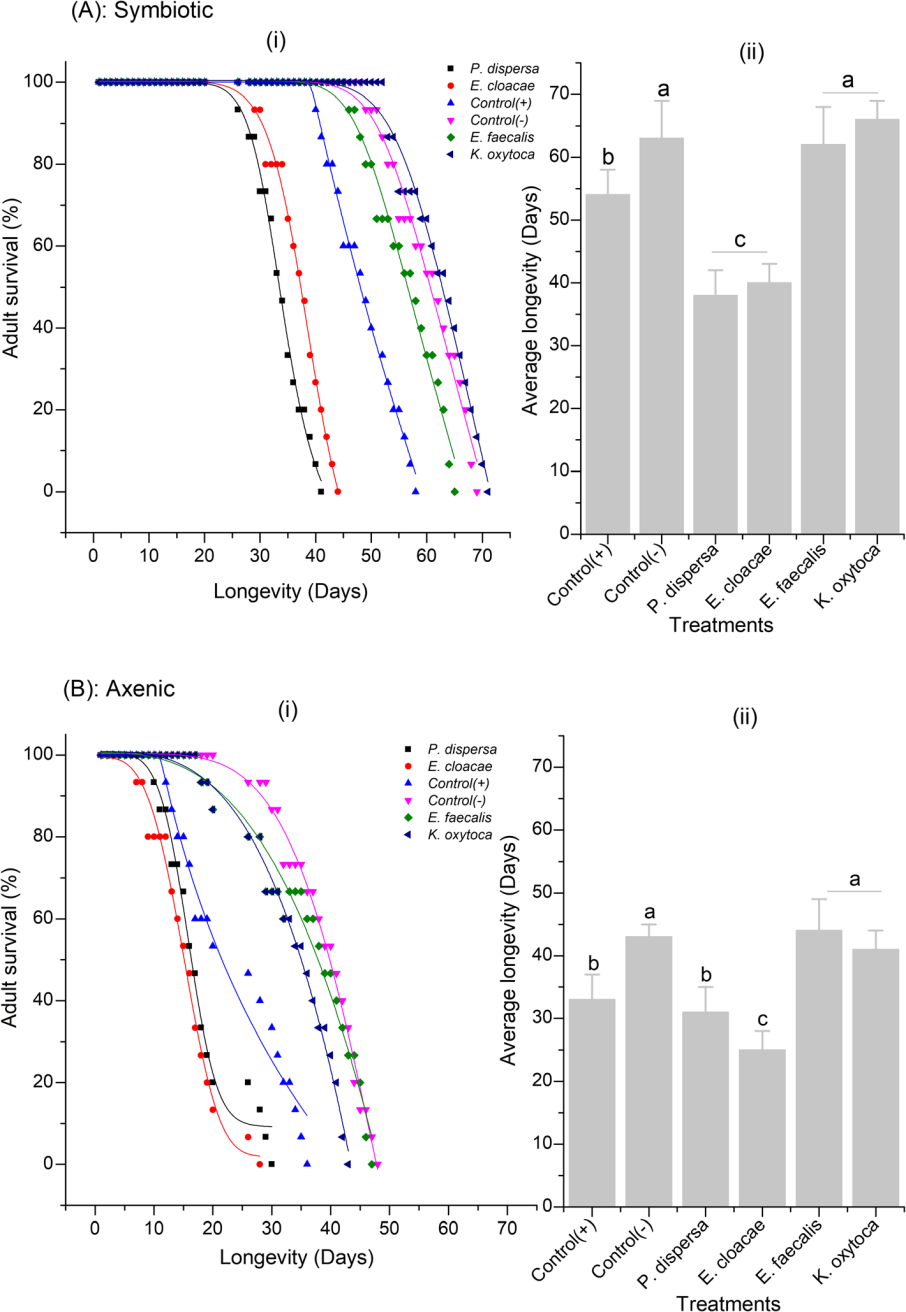
Cumulative survival and average longevity of *B. dorsalis*. Positive control consisted of females maintained under full diets only till death and the negative control consisted of females maintained under sugar diet only till death. (i): daily adult mortality & (ii): average longevity. *P. dispersa* and *E. cloacae* antagonistically regulated the female survival with *E. faecalis* and *K. oxytoca*. From 1 to 7 days, all the flies were protein starved and fed sugar diet only. Mean bars with different letters between treatments are statistically different after Tukey’s post hoc tests at *P* ≤ 0.05

Adult survival was significantly affected by the symbiotic status of flies and diet types (Cox’s Regression Model, HR = 1.47, *P* < 0.0001 and HR = 1.18; *P* < 0.0001, respectively). Overall, symbiotic females lived two-fold longer than the axenic ones, irrespective of the diet consumed (F = 83.637, df = 1, 59, *P* < 0.001) (Fig. 4 A & B). *Enterococcus faecalis* and *K. oxytoca* exerted positive effects on the longevity of symbiotic and axenic female flies. These bacterial isolates significantly extended the female life expectancy by about 14.29% in comparison with the positive control, (Symbiotic: F = 300.946, df = 5, 55, *P* < 0.001 and Axenic: F = 284.746, df = 5, 55, *P* < 0.001) (Fig. 4 A & B). There was no longevity difference between the negative control (in which flies lived longer than in positive control) and the fecundity promoting bacteria (*E. faecalis* and *K. oxytoca*) in both symbiotic and axenic flies (F = 67.381, df = 3, 57, *P* = 0.065 and F = 67.381, df = 3, 57, *P* = 0.127, respectively). Conversely, *P. dispersa* and *E. cloacae* had significantly shortened the longevity of all tested flies by 21.43% compared to positive control (F = 300.946, df = 4, 56, *P* < 0.0001 and F = 284.746, df = 4, 56, *P* < 0.0001, in symbiotic and axenic flies, respectively). Moreover, a paired analysis of full diet and bacterial isolates (*P. dispersa* and *E. cloacae*) revealed a significant interaction between the two factors in shaping the longevity of tested females (χ^2^ = 13.26, df = 4, R^2^ = 0.9841, *P* = 0.001 and χ^2^ = 19.83, df = 4, R^2^ = 0.9889, *P* < 0.001, in symbiotic and axenic flies, respectively) (Fig. 4 A & B).

## Discussion

The relationships between the flies and their gut microbiome are interlaced in complex webs in which gut bacteria provide essential nutrients to the host and enhance its reproductive capacity and survival [1, 37]. The alteration of gut bacteria generally results in the disruption of physiological functions of the host fly. In order to survive and reproduce under such condition, the flies optimally forage on diets which offer higher profitability in terms of nutrient intakes [1, 3]. In a similar experimental setting using symbiotic and aposymbiotic flies, the suppression of gut microbiome by antibiotics treatment disrupted the foraging behavior of the oriental fruit fly *B. dorsalis* and constrained the fly to consume many food droplets at the cost of extending the foraging duration [18]. In our experiment, by creating different feeding environments, we demonstrated how supplementing the normal diet with gut bacteria isolates affected the foraging behavior, diet ingestion and fitness parameters of *B. dorsalis* in a significant manner. Axenic flies (males and females) showed significant preference for the probiotic meal to which they responded faster (compared to full diet) and maximized the diet ingestion to ensure their maintenance and reproductive fitness. Previously, gut bacterial isolates (*Enterococcus cloacae, Citrobacter freundii, Bacillus cereus, Enterobacter, Klebsiella* etc.) were demonstrated to produce chemical substances which attract *Bactrocera dorsalis* and *Bactrocera cucurbitae*, toward available food source [44–46]. In addition to being highly attracted toward the probiotic diets, axenic flies were compelled to ingest as many food drops as possible to become satiated (Fig. 2 A & B). This finding suggests that bacterial isolates may facilitate the access and assimilation of the available nutrients from the diets [32], either by increasing the food palatability or positively modulating digestive enzymes [29]. Previous studies also demonstrated that alterations of gut microbiota may result in the change of feeding behavior and may constraint the host fly to make rational decision toward diets with higher rewards [13, 18] and the use of intestinal bacteria as dietary supplements may help to restore the initial fitness of the host. For example, commensal bacterial isolates *Klebsiella oxytoca* (BD177) was able to reinstate the ecological and foraging fitness of *B. dorsalis* irradiated lines by improving the diet ingestion [18] and increasing food metabolism (haemolymph sugar and amino acid contents) [24].

The amount of ingested food (full diet supplemented with *Pantoea dispersa* and *Enterbacter cloacae*) by the symbiotic flies (which already have bacteria in their gut) and the axenic ones (which do not harbor any of the given bacteria) was similar in foraging experiment (Fig. 2a). This could be due to the ability of bacteria isolates to recolonize their natural habitat in the gut of the axenic flies and revive appetitive behaviors expedited during the bacterial suppression. On the other hand, since the symbiotic flies contain its intact gut microbiota, the effects of these bacterial isolates could be synergistic to the ones already present in the gut of the normal flies. Also, the quantification of the bacteria (four isolates) in fly or fly gut after they have been provided with full or probiotic diets may answer or provide strong clue that those bacteria (not any other factor) impacted the phenotype or affected the foraging behavior observed here.

Supplementing the full diet with *P. dispersa* and *E. cloacae* resulted in an improvement of female fecundity compared to positive control (Fig. 3 A & B). The quality of diets and bacteria were shown to interact together in modulating the fecundity of many fruit flies [37, 47, 48]. The host fly can either use nutrients from the full diets directly to improve its reproduction fitness [28, 29], or simultaneously, the bacterial isolates can use amino acids from the diets to support their own proliferation, before being digested by the host fly and used as additional source of nutrients for eggs production [25, 26]. Conversely, when the diets were supplemented with *E. faecalis* and *K. oxytoca*, the number of eggs laid was reduced by more than 60% in comparison with the positive control, and no eggs were recorded from the negative control in which flies were fed white sugar diet only (Fig. 3 A & B). Two implications can be attributed to these observations. First, *E. faecalis* and *K. oxytoca* may be deleterious by negatively affecting *B. dorsalis* sexual maturity and oogenesis, and the little eggs produced were solely sustained by the full diet. Second, the absence of eggs in sugar fed flies indicates that amino acids residues are primary precursors of eggs production in *B. dorsalis*. Taken together, the association between gut microbiome and diet quality has a nutritional and life history basis [21, 37, 49, 50]. In the same light, the establishment of bacterial isolate (for example, *Acetobacter thailandicus*) in the gut of *Drosophila melanogaster* accelerated the host development and enhance the fertility of the offsprings, and their removal represses the oogenesis in comparison to the normal flies [51, 52].

The nutrient content of diets has significant impacts on adult longevity [53, 54]. For example, the variation of the concentrations of carbohydrate, protein and a phenolic compound (resveratrol) extended the lifespan of *Drosophila melanogaster* [55]. When flies forage in an environment with varying protein availability, they generally make compromises between some fitness parameters based on life history tradeoffs. As such, either they sacrifice the reproduction and prolong their lifespan or maximize energy for reproduction at the cost of shortening their life expectancy. Irrespective of the compromises made along this process, the gut microbiome may come into play to facilitate peptide synthesis or protein metabolism to sustain the host development and survival [21, 49, 50].

The presence of *E. faecalis* and *K. oxytoca* in adult diets extended the *B. dorsalis* lifespan in comparison with the positive control (Fig. 4 A & B). The possible reason could be the ability of these bacterial isolates to reduce biomarkers of physiological and oxidative stresses, and inflammation which are considered as the main cause of early death in flies [56]. Another mechanism of lifespan extension in *B. dorsalis* could be the indirect repression of genes involved in aging pathways by the bacteria [55]. There have been a growing number of studies indicating the ability of intestinal probiotics to extend fly’s life. For example, the inoculation of adult diet with gut bacteria (such as *Enterococcus phoeniculicola* and members of Enterobacteriaceae) prolonged the lifespan of *Ceratitis capitata*, *B. dorsalis* and *D. melanogaster* [32, 56, 57]. However, the incorporation of *P. dispersa* and *E. cloacae* in the full diet resulted in the reduction of lifespan compared to positive control (Fig. 4 A & B). These bacteria might have obstructed the access to nutrients from the full diets by decreasing the food palatability and/or host appetite and promoting the oxidative stress enzymes as previously suggested with *Citrobacter braakii*, *Klebsiella pneumoniae* and *Pseudomonas dispersa* in *B. minax* [58].

## Conclusion

The study of specific functions of gut bacteria on foraging activity and fitness of *B. dorsalis* is elusive to date. Here, we evaluate the effects of four bacterial isolates on the foraging choice and fitness of *B. dorsalis*. Our results show that the axenic flies consistently chose diets inoculated with bacteria to which they responded faster and consumed more droplets than the normal (full) diet. Consequently, diets supplemented with *Pantoea dispersa* and *Enterobacter cloacae* enhanced the female fecundity, while *Enterococcus faecalis* and *Klebsiella oxytoca* enriched diets extended by far the female life expectancy compared to the control. Although further studies are needed to elucidate the molecular mechanisms underlining the symbiont-host interactions (for example, comparative genomics and transcriptomics, microarray, RT-qPCR etc), our results showed to some extent, the potentials of *E. faecalis*, *K. oxytoca*, *P. dispersa* and *E. cloacae* to drive the foraging behavior and alternatively improve lifespan and reproduction of *B. dorsalis*. Since this fly can be controlled by the sterile insect technique (SIT), the intestinal probiotics evaluated in this study could be useful in mass-rearing and longevity extension.

## Methods

### Fly rearing and maintenance

The wild strain larvae of *Bactrocera dorsalis* were collected from infested orange fruits in the experimental orchard of Huazhong Agricultural University (30°4′N and 114°3′ E) (Wuhan, Hubei Province, China) in September 2014 and were reared as previously described [18]. Briefly, the third instar larvae were allowed to exit the fruits, pupate and eclose in sterile sands under controlled laboratory conditions (12:12 light-dark photoperiod; temperature 25 ± 3 °C, and 67 ± 5% relative humidity). The resulting adults were maintained under artificial diet consisting of Tryptone (25 g/L), Yeast extract (90 g/L) (Oxoid LP0021, RG24 8PW, UK), Sucrose (120 g/L), Agar powder (7.5 g/L), Methyl-p-hydroxybenzoate (4 g/L), Cholesterol (2.3 g/L), Choline chlorite (1.8 g/L), Ascorbic acid (5.5 g/L) and 1 L of distilled Water for preparation [18]. Water was provisioned ad libitum in cotton wool. The larval diet consisted of all the above ingredients that were mixed, added with 250 g/L of wheat bran and autoclaved before use [18]. Twenty (20) adult flies aged 8–10 days were removed from the laboratory culture and anesthetized at − 20 °C for 5 min prior to dissection and isolation of individual guts. A culture-dependent technique was employed to isolate gut bacteria (from anesthetized flies) from which, four isolates were later used in bioassays.

### Production of experimental flies

#### Symbiotic flies

Symbiotic flies were collected from the laboratory established colony (as described above). A total of 690 newly emerged flies (1 day old) were fed sugar diet and water for seven days prior to bioassays (to starve them of protein source) using 9 cm Petri dishes presented in cotton wool. One hundred fifty flies were used for foraging tests (75 males and 75 females) (Experiment 1). Three hundred sixty males collected from the lab culture were used to fertilize the 180 females assigned for fecundity and longevity assays (Experiment 2). For Experiment 1, the flies were divided by sex (75 males and 75 females) and separately held in 45x30x30cm cages. Then, individual fly was transferred to a 15x15x15cm cages for bioassays. For Experiment 2, 180 females were separately held in 6 cages of 30 flies each. Then, 60 males (same age) from the lab culture were added in each cage (from the fourth sugar treatment day) to mate with the experimental females. Mating couples (duration ≥10 min) were retrieved and held in a separate cage and later used for bioassays.

#### Axenic flies

Axenic flies were obtained from sterilized eggs (embryos) collected from our lab culture and grown on sterile diets as previously described [51, 59]. Briefly, 300 collected eggs (aged 4 h) were individually surface sterilized twice in ethanol 70% and then rinsed twice in deionized distilled water (ddw), before being immersed in phosphate-buffered saline (PBS) solution for 5 min. The resulting embryos were dechorionated in 2.7% sodium hypochlorite solution for 2 min, and then washed twice in sterile ddw, before being transferred to autoclaved larval diet and allowed to develop for about nine days (to reach the third instar larvae). The third instar larvae were allowed to pupate and eclose in sterile sands under lab conditions (12:12 L: D; 25 ± 3 °C, and 67 ± 5% RH). The axenic state of disinfected embryos was validated by PCR of the 16S rRNA gene on ten individual egg homogenates using universal primers (27F/1459R) and by culturing technique on ten individual egg homogenates using dilution plating of eggs homogenate on agar plate-medium, respectively. The PCR reactions were performed in a programmed thermal cycler under the following conditions: Initial denaturation at 95 °C for 5 min, followed by 30 cycles at 94 °C for 1 min, annealing at 53 °C, 54 °C, 55 °C or 58 °C for 1 min 30 s, 72 °C for 1 min and a final extension at 72 °C for 5 min. Any disinfected sample containing grown colonies or agarose bands were discarded and repeated until no bacterial colonies or DNA bands were seen. The repartition of the number of axenic flies and procedures in both experimental settings (1 and 2) is similar to that of the symbiotic flies as described in the previous section, with the only difference that we used axenic flies here.

#### Insect dissection

The 20 flies previously anesthetized (see section 1 above) were individual washed in 70% ethanol for 2 min and rinsed 3 times in sterile distilled water before dissection. The dissection was carried out aseptically with two pairs of sterilized forceps on a sterilized glass slide spotted with 50 μL of sterile distilled water using a stereomicroscope. The intact guts were individually and separately transferred into Eppendorf tubes containing 750 μL TE buffer (10 mM Tris-Cl, pH 8; 1 mM EDTA, pH 8) and manually crushed with adapted pestle. Homogenized gut suspensions were serially diluted by 10^− 4^–10^− 8^, 50 μL of which were plated onto Luria Bertani (LB) agar media (Table 1) and incubated at 37 °C for 24–48 h. After the incubation, the representative bacteria colonies were randomly pooled based on the size, color, opacity and morphology of each colony. The pre-selected colonies were purified through repeated sub-culturing before being used for DNA extraction and sequencing or preserved in glycerol at − 80 °C in 50% glycerol (v/v) for future use. The whole dissection procedures were performed in a laminar flow hood to avoid aerial contamination.

**Table 1:** Ingredients and preparation of the standard Luria Bertani (LB) agar media

Ingredients		Amounts (g)	Preparation procedures
1	Yeast extract	2.5	Ingredients 1–4 were put in an Erlenmeyer containing 250 mL of distilled water and the solution was mixed with a magnetic stirrer. Then distilled water was added to total volume of 500 mL and transferred to 1 L flask. The liquid was autoclaved for 20 min at 115 °C and let to cool at ~ 55 °C before pipetting 25 mL onto each petri dish plate.
2	Tryptone	5	
3	NaCl	5	
4	Agar powder	7.5	
5	Water	500	

Note: The preparation of LB broth follows the same procedures but without agar powder

#### Bacterial DNA extraction

The DNA was extracted following the CTAB (Cetyl TrimethylAmmonium Bromide) method. Briefly, single purified colony was cultured in LB broth for ~ 7 h. 1.5 mL of bacterial suspensions were centrifuged at 10,000 rpm for 10 min and the recovered pellets were re-suspended in 557 μL of TE buffer. 10 μL of lysozyme (5 mg/ml) was added to the suspension and incubated at 37 °C for 20 min. Then, 3 μL proteinases K (20 mg/mL) and 30 μL SDS (10%) were respectively added and incubated again at 37 °C for 40 min, afterward 100 μL of NaCl (5 mol/l) and 80 μL of CTAB/NaCl were added to the solution and incubated again at 65 °C for 10 min. Then, Phenol/chloroform/ isoamyl alcohol (25:24:1) was finally added to the upper phase solution and centrifuged at 13,400 g for 4 min. Finally, Isopropyl alcohol was added to precipitate the DNA pellets which were later rinsed in 70% ethanol, re-suspended in TE buffer and kept at − 20 °C until use for PCR analysis.

#### Polymerase chain reactions (PCR)

PCR reactions of the 16S rRNA gene were performed using the bacterial universal primers 27F:5′-AGAGTTTGATCMTGGCTCAG-3′ and 1492R: 5′-GGTTACCTTGTTACGACTT-3′. A total volume of 50 μL of PCR reactions containing 1 μL of DNA template, 1 μL of F/R primers, 5 μL of High Fidelity DNA buffer (× 10), 4 μL of dNTPs (2.5 mM), 1 μL of Hifi DNA polymerase and 38 μL of deionized distilled water was prepared. The amplification was carried out in a programmed thermal cycler under the following conditions: an initial denaturation step of 95 °C for 5 min followed by 34 cycles of denaturation at 95 °C for 1 min, annealing at 55 °C for 1 min, an extension phase of 72 °C for 1 min and a final extension at 72 °C for 10 min. The PCR amplicons were analyzed by electrophoresis on a 1% agarose gel and visualized under UV light after staining with ethidium bromide. The target band (1.5 kb) was purified with a DNA gel extraction kit (Axygen, China). The purified DNA samples were sequenced using Illumina sequencing technology (Novogene, China) and identified by comparison with the most similar sequences from NCBI nucleotide database using the megablast algorithm (http://blast.ncbi.nlm.nih.gov/) (Table 2).

**Table 2:** Identity of gut bacterial isolates from *Bactrocera dorsalis* based on 16S rRNA gene sequences

Isolate labels	Sequence length (bp)	Best tblastn hit species	GenBank accession No.	Identity (%)	Family
BDF-1	1399	*Enterobacter cloacae*	CP030347.1	99	Enterobacteriaceae-
BDF-2	1467	*Enterococcus faecalis*	MG543815.1	99	Enterobacteriaceae+
BDF-3	1452	*Klebsiella oxytoca*	NR_114152.1	99	Enterobacteriaceae-
BDF-4	1433	*Pantoea dispersa*	AY227805.1	99	Enterobacteriaceae-

### Preparation of experimental diets

#### Full and sugar diet

A total of two different diets were prepared: a full diet (F) containing all amino acids (essential and non-essential), sucrose, and minerals required for an optimal maintenance and reproductive development of adult flies [29]; a sugar diet consisting of 60% sucrose and minerals used to maintain flies for seven days of protein starvation (Table 3). The diet compositions and preparation procedures were done as previously described [29] and filtered before use.

**Table 3:** Nutrient composition of experimental diets

Ingredients	Components	Contents (mg)	
		Full diet	Sugar
Essential amino acids	L-arginine	50.45	
	L-histidine	21.54	
	L-isoleucine	26.64	
	L-leucine	51.02	
	L-lysine	27.78	
	L-methionine	13.04	
	L-phenylalanine	33.44	
	L-threonine	25.51	
	L-tryptophan	13.60	
	L-valine	37.41	
Non-essential amino acids	L-alanine	36.85	
	L-aspartic acid	53.28	
	L-aspartic acid	19.27	
	L-glutamic acid	185.36	
	Glycine	42.51	
	L-proline	58.95	
	L-serine	36.85	
	L-tyrosine	22.67	
Minerals and salts	FeSO_4_	2.50	2.50
	MnSO_4_	0.63	0.63
	ZnCl2	0.63	0.63
	CuSO_4_	0.31	0.31
	MgSO_4_	20.00	20.00
	KH_2_PO_4_	84.65	84.65
	Ca(H_2_PO_4_)2	10.00	10.00
	KCl	117.00	117.00
	NaCl	45.00	45.00
	White sugar	10,000.0	10,000.0
	DDW	50,000.00	50,000.00

*F* Full diet contains all amino acid, *DDW* Deionized distilled water

#### Probiotic diets

Four bacterial isolates (*Pantoea dispersa*, *Enterobacter cloacae, Enterococcus faecalis* and *Klebsiella oxytoca*) (Table 2) were separately grown in LB broth (Table 1) and centrifuged at 10,000 rpm for 5 min. The harvested pellets were washed twice and resuspended in sterile distilled water. The concentration of bacteria in the solvent was adjusted to 0.5 optical density (OD) at 550 nm wavelength using a spectrophotometer (Eppendorf AG, Germany) [32]. 500 μL of each bacterial suspension was added to 50 g of full diets (treatment groups) while 500 μL of sterile distilled water only was respectively added to full and sugar diets (positive and negative controls, respectively). Two Petri dishes seeded with 5 drops of 5 μL volume of full and probiotic diets (randomly displayed in Petri dishes), respectively, were used for the foraging experiment. The flies assigned for fecundity and longevity assays were fed with 1 mL daily of each experimental diet presented in petri dishes seeded with autoclaved cotton wool.

### Experimental procedures

#### Experiment 1: foraging assays

**Fig. 5:**
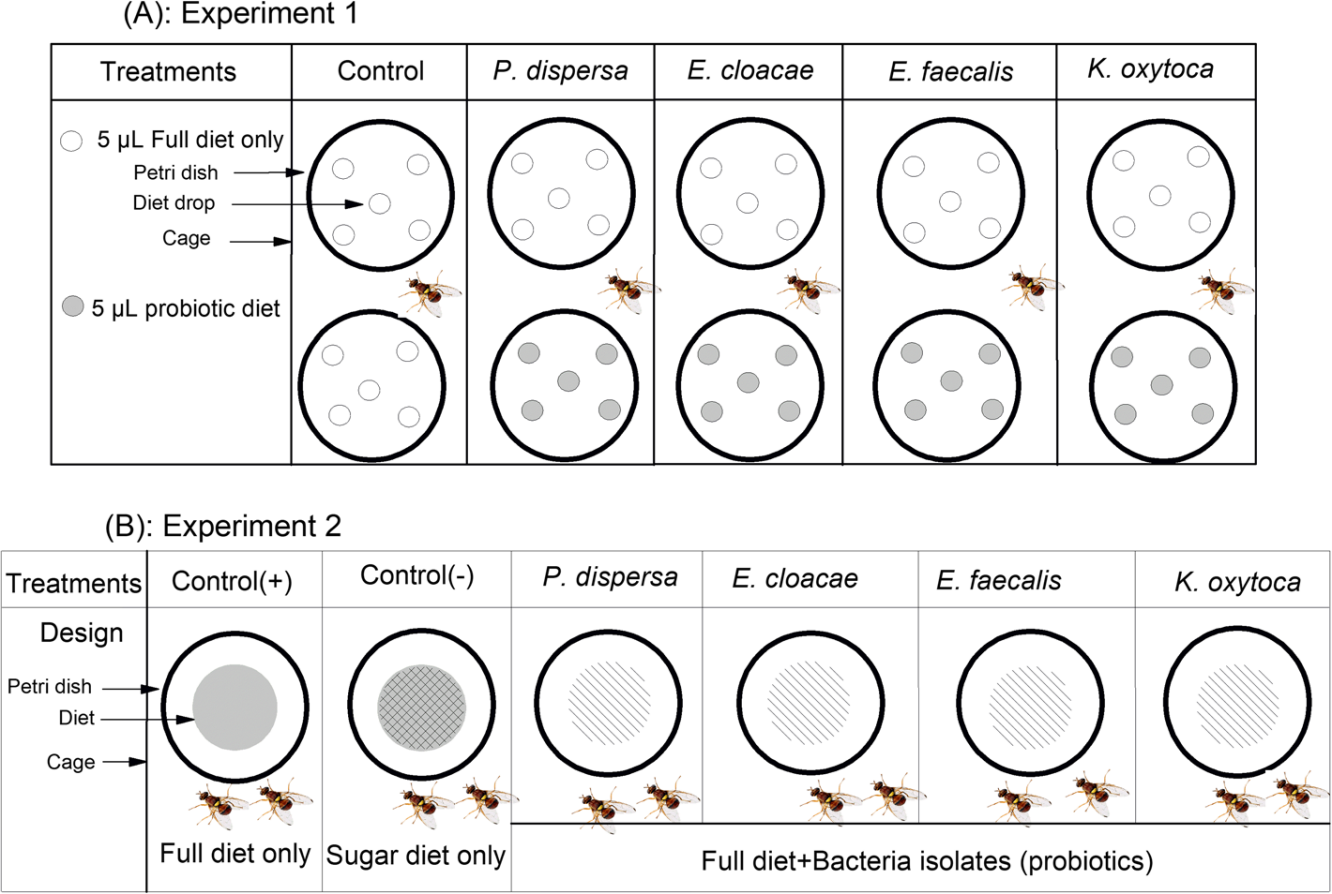
Experimental design for foraging assays (**a**) and fitness parameters assays (fecundity and longevity) (**b**). The full diets contain all the amino acids, and the probiotic diets contain full diet + bacteria isolate

Following the seven day preparatory period during which flies were fed only sugar (as described above), individual fly was transferred to a 15x15x15cm cage and allowed to acclimatize for 20 min before introducing simultaneously a pair of petri dishes containing combinations of Full diet and bacteria supplemented diets at similar volumes and densities (Fig. 5a). Five treatment groups were set up representing the four bacterial isolates and a control group (Fig. 5a). Female and male flies were tested separately and to motivate their foraging responses, they were all starved for 24 h before experimental trials. Each observation event was replicated 15 times, males and females, symbiotic and axenic flies, representing a total of 300 observation events (15 replicates × 2 symbiotic status × 2 sexes × 5 treatments). Each replicate consisted of observing the protein starved individual male or female (symbiotic and axenic) for an hour and collecting data on latency (time from diet exposure to the initial landing), the number of flies which landed on a food drop, the choice of diet made and the number of drops consumed. All the data collected were then analyzed within and between treatments and symbiotic status.

#### Experiment 2: fecundity and longevity assays

A total of 720 newly emerged 1 day old flies were preselected for this experiment: 360 females (180 symbiotic and 180 axenic) and 360 symbiotic males exclusively (60 males × 6 treatments). Symbiotic and axenic flies were separately held in six 45x30x30cm cages of 90 flies, each containing 30 females and 60 males, respectively (1:2 proportion). Symbiotic and axenic flies were starved for 24 h to obtain homogenous populations before being separately fed with autoclaved sugar diet and water ad libitum (soaked in cotton wool presented in Petri dishes) for seven days. Thirty individual mating couples, symbiotic and axenic were immediately retrieved from each cage, individually held in 15x15x15cm cages and the mating duration was evaluated. Only a mating time ≥ 10 min was considered, otherwise the couple was returned to initial cages. Six treatment groups containing 60 flies each (30 couples) were set up, representing the different types of diet with which mated couples were maintained (Fig. 5b). At seventh day, the sugar diet was replaced by either full diets (positive control) or probiotic diets (treatments with each of the four bacterial isolates), or maintained under the same sugar diets (negative control) (Fig. 5b). Agar medium inoculated with orange juice served as oviposition substrate. The female fecundity was evaluated by counting the daily number of eggs laid by individual female maintained in 100 mL transparent plastic cups throughout the female life expectancy. A yellow circular paraffin residue (Ø ≈ 6 cm, width ≈ 1 cm) was placed at the bottom of each cup and used as oviposition substrate to collect eggs daily [29]. The female survival was assessed by daily cage inspection and counting of dead flies till their complete death in all treatment groups. The data were pooled and analyzed within and between treatments and symbiotic status.

### Statistical analysis

A one-way analysis of variance (ANOVA) was performed to separately test the effects of symbiotic status and diet types on the foraging behavior (landing latency and diet consumption) (male and female), female fecundity and longevity. All data were tested for homogeneity of variances using Levene’s tests and only data on the effects of diet types on female fecundity were log transformed. The non-parametric test (Kruskal-Wallis H) was used when ANOVA assumptions were violated (for example, data on survival, F = 18.68, df = 5, 45, *P* < 0.0001). To assess the responses of experimental flies (symbiotic and axenic), marginal means of all flies which landed on either full diet or probiotic diet were analyzed using chi-square test, Student’s t-test was used to determine the statistical difference between both experimental diets and their corresponding latencies (irrespective of treatments) were pooled and computed by ANOVA. The one-way ANOVA was used to analyze the food consumption between males and females using OriginPro software version 8.5.1 and IBM SPSS 20.0 software. To determine the importance of factors that shape the foraging behavior of *B. dorsalis*, variables of overall response and latency were analyzed using the ordinary least squares regression model (IBM SPSS 20.0 software) with sex, symbiotic status, diet types and treatments (see Fig. 5a) as effects. Similarly, to determine the crucial factors that shape the survival, percentage of daily living flies were analyzed using the Cox’s regression model (SPSS 20.0 software) with diet types and symbiotic status as effects. The Pearson chi-square test was used to assess the association between the full and probiotics diets in modulating the foraging behavior, fecundity and longevity of experimental flies. Multiple comparisons between treatments were based on Tukey’s post hoc tests at *P* ≤ 0.05. The IBM SPSS software 20.0 (SPSS Inc., Chicago, IL, U.S.A.) was used to analyze all datasets and expressed as the means with standard errors (SE), except data on the overall responses. OriginPro software version 8.5.1 was used to draw curves and graphs.

## Data Availability

The 16S rRNA gene sequences of bacterial isolates *E. faecalis*, *K. oxytoca*, *P. dispersa* and *E. cloacae* used in this study are submitted to NCBI GenBank (accessions MK764696, MK749918, MK749919 and MK749920) and to dryad following the link: https://datadryad.org/review?doi=doi:10.5061/dryad.tk3k002. The sequences of other bacterial isolates from this study can be obtained through the accessions MK764697, MK764698, MK764699, MK764700, MK764701, MK764702, MK764703, MK764704, MK764705, MK764707, MK764708, and MK764709.
